# A Tree-Centered Approach to Assess Impacts of Extreme Climatic Events on Forests

**DOI:** 10.3389/fpls.2016.01069

**Published:** 2016-07-21

**Authors:** Ute Sass-Klaassen, Patrick Fonti, Paolo Cherubini, Jožica Gričar, Elisabeth M. R. Robert, Kathy Steppe, Achim Bräuning

**Affiliations:** ^1^Forest Ecology and Forest Management Group, Wageningen UniversityWageningen, Netherlands; ^2^Landscape Dynamics Unit, Swiss Federal Institute for Forest, Snow and Landscape Research WSLBirmensdorf, Switzerland; ^3^Department of Forest Yield and Silviculture, Slovenian Forestry InstituteLjubljana, Slovenia; ^4^CREAFCerdanyola del Vallès, Spain; ^5^Laboratory of Plant Biology and Nature Management, Vrije Universiteit BrusselBrussels, Belgium; ^6^Laboratory of Wood Biology and Xylarium, Royal Museum for Central AfricaTervuren, Belgium; ^7^Laboratory of Plant Ecology, Department of Applied Ecology and Environmental Biology, Faculty of Bioscience Engineering, Ghent UniversityGhent, Belgium; ^8^Department of Geography and Geosciences, Friedrich-Alexander-University Erlangen-NurembergErlangen, Germany

**Keywords:** climate change, future forests, tree, mechanistic understanding, structure-function relationships, long-term monitoring, intra-annual resolution, resilience

## Introduction

A major task of our society is to manage forests in a way that their resources are preserved to meet future generation needs (Forest Europe et al., 2015). Current scenarios of climate change effects are making this task extremely challenging (Kirilenko and Sedjo, 2007). Climate shifts will impact forest vitality and affect goods and services forests provide, including carbon sequestration and climate change mitigation (IPCC, 2014). To guide sustainable forest management, forest researchers are asked to provide concrete answers about forest resilience in response to expected climatic trends, and extreme climatic events (Lindner et al., 2014). This is not an easy task, because responses of trees and forest ecosystems to environmental conditions are often non-linear and moreover vary on spatial and temporal scales (Smith, 2011; Anderegg et al., 2012; Reichstein et al., 2013). For instance, although drought is one of the most frequent and widespread climatic extremes affecting forests worldwide (e.g., Allen et al., 2010), the assessment of its impact on future forests is currently under intense debate. Mechanisms behind tree growth and mortality are complex (McDowell et al., 2008, 2011; Fatichi et al., 2014; Anderegg et al., 2015; Meir et al., 2015). Besides strength or frequency of external factors, such as extreme events, also the tree's ability to resist and recover is relevant, which, in turn, is largely determined by intrinsic factors such as the tree's life stage, life history, and genetic characteristics.

In this paper, we advocate for a tree-centered approach. By providing an improved mechanistic understanding of physiological and growth responses of trees growing under various conditions we can define the tree's capacity to respond to external stress factors. This concept can valuably contribute to the debate on how to shape future forests toward resilient forest ecosystems.

## A tree-centered approach

Current spatiotemporal simulations on future forest growth responses to changing climate conditions are performed with dynamic global vegetation models (DGVMs; Wullschleger et al., 2014). These models—usually generalizing tree species as plant functional types (PFTs)—provide valuable descriptions of the evolution of natural vegetation at a grid cell level under several climate scenarios. Such approaches are powerful in assessing growth responses related to the interaction between vegetation and atmosphere (including anthropogenic impact). However, although first steps toward representation of tree species, size classes, and forest structure in a DGVM were recently made (e.g., Naudts et al., 2016) they often lack to explain the variability between and within species, and often do not adequately explain growth responses (Fatichi et al., 2014) under varying site conditions, and to climatic extremes (Anderegg et al., 2015). These aspects are however extremely relevant to evaluate plasticity of tree individuals and tree species and the resilience of forests under changing climatic conditions, especially considering changing frequencies and intensities of climatic extremes (Reyer et al., 2013).

The tree-centered approach proposed here considers the individual tree as main source of information for understanding variability in growth responses. Comprehensive investigations using well-selected trees growing under different environmental conditions foster a better understanding of projected large-scale forest responses to changing climate. In comparison to generalizing approaches using PFTs, the tree-centered approach yields information with less spatial coverage but with the potential to convey more details on specific tree responses to a given climatic factor. This knowledge complements other approaches and can for instance support forest managers in tree species and/or provenance selection to better prepare specific forest stands to cope with expected challenges.

## Four important elements

The incentive for the tree-centered approach is gaining a process-based understanding on tree responses to changing environmental conditions on temporal scales varying from short-term responses to climatic extreme events to long time periods matching the life cycles of tree populations. This can be achieved through an ensemble of observational studies on a selection of trees from different species and life histories, growing in diverse settings (forest types, species composition, successional stages, management regimes), and exposed to different climates and extreme climatic events. Establishing such a model framework requires the following elements:

- In-depth understanding of causal processes occurring within the tree in response to environmental changes. The cascade of physiological and growth responses is assessed by an integration of real-time observations of physiological and structural growth responses.
- Assessment of the link between tree structure and function. This allows for evaluation of the short-term impact of extreme events on tree functioning as a consequence of resulting structural changes in tree morphology as well as wood and bark structure.
- A long-term temporal perspective to verify the link between a specific event and related responses. Here we take advantage of the fact that trees rigorously archive growth responses within their datable annual tree rings. Dendrochronology provides this necessary historical perspective for quantifying resilience by assessing impacts of past events.
- Comparative studies in selected sites experiencing extreme events, long-term manipulation studies, and experiments, including e.g., provenance trials are finally necessary to test and validate the model framework to conditions expanding even far outside today's natural range.

## Implementation of the tree-centered approach

**Figure 1:**
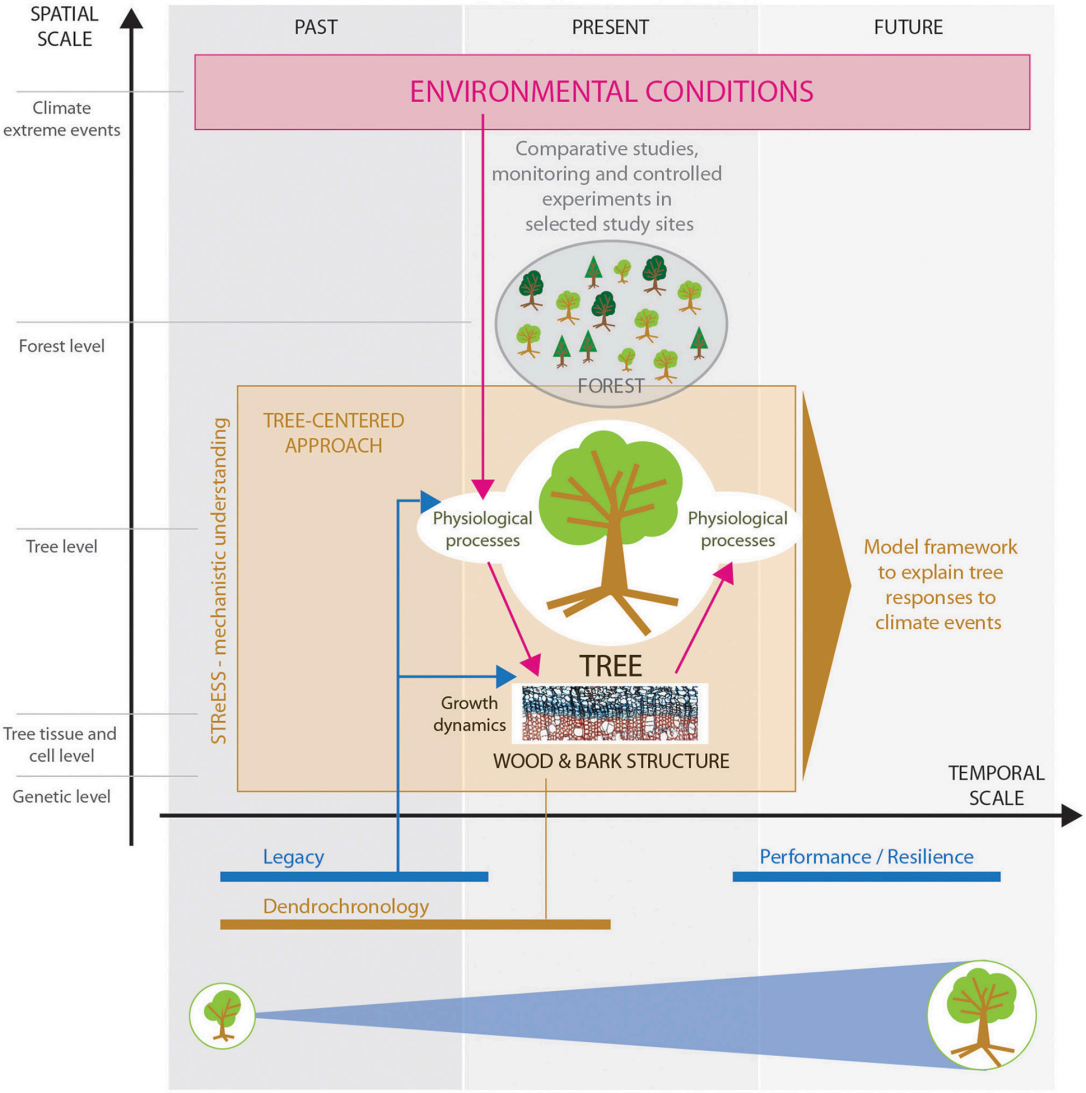
**Schematic of the tree-centered approach applied by STReESS to enhance mechanistic understanding**. The approach consists of the following interlinked elements: (1) Environmental conditions affect physiological processes, which drive growth dynamics, and results in specific wood and bark structure. (2) Quantity and structure of newly formed wood as assessed by dendrochronology affects whole-tree physiological functioning and performance. (3) Legacy of past environmental conditions and extreme events is imprinted in wood structure, influencing tree functioning, and todays' tree performance. This is important to assess tree resilience ability. (4) Selection of comparative studies, monitoring, and controlled experiments allow model testing and validation in specific contexts. All four elements are important to assess non-linear trigger-response relations and enable to create a model framework to explain and evaluate tree responses to climate events.

The COST Action STReESS—a 4-year European framework initiative to promote networking among researchers of several plant-research disciplines to *study tree responses to extreme events*—is demonstrating the potential of such an integrated bottom-up approach. Starting from an enhanced understanding of the physiological processes behind wood formation, the STReESS Action established a modular process-based approach, which will eventually result in a model framework for explaining tree responses to climate extremes (Figure 1).

The COST Action STReESS contributed to the following main interlinked elements of the concept along the causal path: environmental trigger—structure—function—performance.

### Long-term high-resolution monitoring

Efforts performed for long-term high-resolution monitoring of tree physiology, tree growth together with contemporary site, and climatic factors, allows quantifying causal relationships between external triggers and tree physiological and growth responses (Steppe et al., 2015, 2016). Knowledge gained from such real-time measurements has resulted in process-based plant models in which the mechanisms underlying diel water and carbon transport and their tight coupling have been integrated (see review by De Swaef et al., 2015). In addition, worldwide xylogenesis and dendrometer databases have been compiled during the STReESS Action to assess global response patterns to various specific climate and site conditions (Rossi et al., 2013) and to gain insight into processes involved in wood formation (Cuny et al., 2014, 2015; Steppe et al., 2015).

### Linking structure to function

Linking structure to function is vital to understand the impact of climate-caused changes on wood formation dynamics and wood structure, which strongly influences the water, and carbon household and determines actual, and future tree survival chances and growth performance. Recent studies have highlighted that tree morphological properties and related e.g., hydraulic safety properties (Delzon and Cochard, 2014) vary within individuals, among provenances and species, or along environments and stress gradients. Relevant parameters include phloem to xylem ratio (Gričar et al., 2015; Jyske et al., 2015) and connection (Pfautsch et al., 2015), as well as xylem and phloem-cell characteristics in stems (Anfodillo et al., 2012; Olano et al., 2013; Carrer et al., 2015; Gričar et al., 2016), branches (Salmon et al., 2015), and roots (Brunner et al., 2015). Such characteristics reflect functional adjustments in the tree's structures in response to changing environmental conditions. In turn, these adjustments also form a legacy by influencing future tree performance and hence reflecting the acclimation capacity of trees and tree species (Lachenbruch and McCulloh, 2014; Rosner et al., 2016a,b; Sterck et al., 2016; Anfodillo et al., in review).

### The long-term perspective

The continuous adjustments in wood structure are permanently stored in tree rings either as annual variations in wood-anatomical characteristics, such as cell-wall thickness, cell size, or tissue percentage, or in case of extreme climatic events, as obvious wood-anatomical markers (Battipaglia et al., 2016; Bräuning et al., 2016). These characteristics enable the use of dated tree rings to reconstruct how trees have been growing and functioning in the past (Fonti and Jansen, 2012), and consequently reflect the resilience and acclimation strategies of trees in a changing climate (e.g., Breda et al., 2006). Typical cases of wood-anatomical markers considered in the STReESS action are flood rings (Copini et al., 2016), missing rings and dark rings (Novak et al., 2016), and intra-annual density fluctuations (IADFs). IADFs comprise an abrupt change in wood density in a given tree ring (Nabais et al., 2014; Campelo et al., 2015) and have been demonstrated to hold valuable high-resolution information on the timing of past droughts in Mediterranean conifers (Battipaglia et al., 2016; De Micco et al., 2016; Zalloni et al., 2016). Recently, these approaches became increasingly applicable due to methodological advances in efficiently quantifying cell-lumen size, cell-wall thickness or specific cell types as resin canals, and parenchyma cells (von Arx and Carrer, 2014; von Arx et al., 2016). The enormous potential of wood-anatomical characteristics and markers lies in the information they provide on the exact timing of climatic constraints (Fonti et al., 2010; Rathgeber et al., 2016) and in the possibility to evaluate consequences that these constraints have for xylem and phloem functioning. This creates the bridge between the elements 1 and 2 and allows to uniquely integrating a long-term functional perspective on the current assessment of tree-growth responses to the environment.

### Field studies, provenance trials, manipulation experiments

The limit of spatial coverage in the high-resolution monitoring approach (element 1) together with the need for testing the effect of future climate-change scenarios on tree performance can be waged by comparative field and experimental studies to target specific species, provenances, or environmental conditions. De Luis et al. (2014) illustrated the potential of using tree-ring networks across species distributions to assess local adaptation and plasticity of *Pinus halepensis* in the Mediterranean basin. Another approach is a trans-European cross experiment where drought mortality-resistance of European beech provenances has been related to water availability and to the origin of the beech seedlings (Pšidová et al., 2015; Bolte et al., 2016). Hence, provenance trials have revealed a genetic control of wood structural properties (Eilmann et al., 2014; Nabais et al., in review), although investigations of other tree species are needed to fully evaluate the importance of genetic preadaptation to future climatic conditions. Such kinds of studies prove the added value of targeting specific situations, e.g., natural conditions or manipulated experiment, for developing, and validating the model framework.

### Integration into a model framework

The four elements can be captured and integrated into process-based tree models. The first steps of this integration have already been achieved. For example, diel stem-size variations and sap-flux densities combining real-time and high-resolution measurements of tree functioning under on-site environmental conditions have allowed to link environmental triggers (climate events) with the resulting tree growth and performance (e.g., Steppe et al., 2006; De Schepper and Steppe, 2010; Hölttä et al., 2010; Schiestl-Aalto et al., 2015). This means that instant responses of a tree to a drought or a heat wave (Teskey et al., 2015) can be readily assessed, and changes in its water and carbon budget quantified. Effort still needs to be invested to implement parameterizing of long-term climate-growth responses or the peculiarity of species and proveniences into the existing models to finally come up with estimates for tree plasticity, acclimation potential of tree species, and ultimately resilience of forests.

## Conclusion and perspective

There is a fundamental difference between generalized PFT-based approaches (i.e., DGVMs) and tree-centered approaches. While PFT-based approaches perform spatially explicit “scenarios” of future global responses, the interdisciplinary process-driven tree-centered approach has potential to also provide practical support for local management decisions based on a solid understanding of tree functioning under specific site conditions. The COST Action STReESS has improved our understanding on the variability of responses to climate trends and extreme events. After 4 years of collaboration, the consortium has collected indications of the usefulness of such an integrated approach with continuous “methodological” development, and creativity. Through the integration of monitoring studies (e.g., time series of dendrometers, wood formation, and forest inventories), dendrochronological approaches, manipulation experiments (e.g., induced drought stress), and process-based models (e.g., at the cell, plant, or vegetation level) there is potential to collect valuable characterization to build process-based tree models accounting for variability between species, provenances, sites, and climatic events. This will contribute to unraveling a large set of yet unanswered questions related to processes of tree mortality (e.g., McDowell et al., 2011) or (mal)-adaptation (e.g., Martinez-Meier et al., 2008). Such a process-based approach will help to reduce uncertainty on tree performance under future environmental conditions.

Actual plans include the extension of the twittering-tree network for further development of a near real-time detection of tree processes and environmental impacts (Steppe et al., 2016) as well as the extension of global data networks and harmonization of protocols for high-resolution growth measurements (e.g., dendrometer, xylogenesis).

Despite still many implementation challenges ahead, we believe that the tree-centered approach offers an additional opportunity to assess forest management sustainability at the profit of the whole society.

## Author contributions

All authors developed the concept and structure of the manuscript. USK and PF wrote the first draft of the manuscript. EMRR developed and designed the figure in cooperation with PF and USK. AB, EMRR, KS, JG, and PC authors accomplished and checked the first version and read and approved the submitted version.

### Conflict of interest statement

The authors declare that the research was conducted in the absence of any commercial or financial relationships that could be construed as a potential conflict of interest.
